# Supplementary material for: Patient education and extracorporeal membrane oxygenation preferences of patients and providers in COVID care
Source: PLoS One. 2024 Aug 13;19(8):e0297374. doi: 10.1371/journal.pone.0297374 (PMC11321548; doi:10.1371/journal.pone.0297374)
Supplement: S1 Table — (DOCX) [file pone.0297374.s001.docx]

**eTable. Survey about Participating Patient and Provider Knowledge, Attitudes, and Preferences about Extra Corporeal Membrane Oxygenation (ECMO):**

The COVID19 pandemic has highlighted the importance of Advanced Care Planning and end-of-life (EOL) care. About 1 in 3 Americans have advanced directives for EOL care if they become seriously ill or unable to make health care decisions. We want to obtain a better understanding of knowledge and attitudes about aggressive treatment during the COVID19 pandemic.

This survey will help us understand the differences between the preferences of the general public and that of healthcare providers with regards to serious illness and EOL care discussions. Please respond to all questions.

*Knowledge questions:*

ECMO:

1. Is a kind of life support that can improve the amount of oxygen in patients
2. Is a medical process that removes blood from one patient and donates it to another person
3. Is a blood test to diagnose patients with COVID19
4. Not sure/Do Not know

Which of the following describes a benefit of ECMO?

1. Assists patients with irreversible lung damage
2. Allows injured lungs to rest while they recover
3. Decreases oxygen delivery to the lungs
4. Not sure/Do Not know

Which of the following describes a risk of ECMO?

1. Heart Disease
2. Cancer
3. Stroke
4. Not sure/Do Not know

Which of the following represents a possible complication of ECMO?

1. Temporary need for dialysis
2. Hepatitis A from a blood transfusion
3. Down Syndrome
4. Not sure/Do Not know

*Preference questions:*

1. Jane is 35 years old and lives at home by herself independently. She recently became sick with COVID19 and is currently in the hospital with a breathing tube. She displays no improvement after 5 days on a breathing machine.

To what extent do you agree that this patient should be considered for ECMO?

- 1. Strongly Disagree
  2. Disagree
  3. Agree
  4. Strongly Agree

1. Jane is 35 years old and lives at home by herself independently. She takes 3 pills a day for diabetes and high blood pressure. She recently became sick with COVID19 and is currently in the hospital with a breathing tube. She displays no improvement after 5 days on a breathing machine.

To what extent do you agree that this patient should be considered for ECMO?

- 1. Strongly Disagree
  2. Disagree
  3. Agree
  4. Strongly Agree

1. Jane is 35 years old and because of weakness in her arms and legs, she needs an aide to help her bathe/dress. She recently became sick with COVID19 and is currently in the hospital with a breathing tube. She displays no improvement after 5 days on a breathing machine.

To what extent do you agree that this patient should be considered for ECMO?

- 1. Strongly Disagree
  2. Disagree
  3. Agree
  4. Strongly Agree

1. Jane is 35 years old and because of weakness in her arms and legs, she needs an aide to help her bathe/dress. She takes 3 pills a day for diabetes and high blood pressure. She recently became sick with COVID19 and is currently in the hospital with a breathing tube. She displays no improvement after 5 days on a breathing machine.

To what extent do you agree that this patient should be considered for ECMO?

- 1. Strongly Disagree
  2. Disagree
  3. Agree
  4. Strongly Agree

1. Jane is 65 years old and lives at home by herself independently. She recently became sick with COVID19 and is currently in the hospital with a breathing tube. She displays no improvement after 5 days on a breathing machine.

To what extent do you agree that this patient should be considered for ECMO?

- 1. Strongly Disagree
  2. Disagree
  3. Agree
  4. Strongly Agree

1. Jane is 65 years old and lives at home by herself independently. She takes 3 pills a day for diabetes and high blood pressure. She recently became sick with COVID19 and is currently in the hospital with a breathing tube. She displays no improvement after 5 days on a breathing machine.

To what extent do you agree that this patient should be considered for ECMO?

- 1. Strongly Disagree
  2. Disagree
  3. Agree
  4. Strongly Agree

1. Jane is 65 years old and because of weakness in her arms and legs, she needs an aide to help her bathe/dress. She takes 3 pills a day for diabetes and high blood pressure. She recently became sick with COVID19 and is currently in the hospital with a breathing tube. She displays no improvement after 5 days on a breathing machine.

To what extent do you agree that this patient should be considered for ECMO?

- 1. Strongly Disagree
  2. Disagree
  3. Agree
  4. Strongly Agree

Thank you for completing the survey questions related to ECMO. We would like you to complete the following demographic questions so we can have a better sense of who you are. Please answer all the following questions.

*Demographic questions:*

1. Do you describe yourself as a man, a woman, or in some other way?
   1. Man
   2. Woman
   3. Some other way-write in
2. What is your age at the time of taking this survey?
   1. 50-60 years
   2. 60-70 years
   3. 70-80 years
   4. 80-90 years
   5. >90 years
   6. Prefer not to answer
3. What is your race? (select all boxes that apply)
   1. White
   2. Black, African American
   3. American Indian or Alaska Native
   4. Asian Indian
   5. Chinese
   6. Filipino
   7. Japanese
   8. Korean
   9. Vietnamese
   10. Pacific Islander
   11. Some other race: print race
4. Are you of Hispanic, Latino, or Spanish origin?
   1. No
   2. Yes
5. What is the highest degree or level of education you have completed?
   1. High School Diploma or less
   2. Bachelor’s Degree
   3. Master’s Degree
   4. Doctorate Degree (Ex: Ph.D., MD, J.D)
   5. Trade/technical/vocational School
   6. Prefer not to say
6. What is your current marital status?
   1. Now married
   2. Widowed
   3. Divorced
   4. Separated
   5. Never married
   6. Prefer not to say
7. Has anybody close to you (friend, spouse, partner, family member etc.) has been hospitalized with COVID?
   1. Yes
   2. No

Thank you for completing this survey
