# Supplementary material for: Patient education and extracorporeal membrane oxygenation preferences of patients and providers in COVID care
Source: PLoS One. 2024 Aug 13;19(8):e0297374. doi: 10.1371/journal.pone.0297374 (PMC11321548; doi:10.1371/journal.pone.0297374)
Supplement: Attachment — Submitted filename: ResponseTOreviewers_REVISION.docx [file pone.0297374.s002.docx]

**Response to Reviewer Comments on PONE-D-23-01032R1**

Reviewer #2: None

Reviewer #3:

1) Ethan D. Borre and the coauthors present a interesting work, which could have value to readers of PLOS ONE and colleagues worldwide if it was again comprehensively revised. While the introduction and material and methods are adequately presented, this is unfortunately not at all the case for the results and discussion.

*Response: We appreciate the positive comments about this manuscript and have revised the manuscript as suggested by comments below.*

2) Far too few results are presented in a much too short form. If the authors want to publish the manuscript in such a prestigious journal, it must be urgently revised.

*Response: The reviewer’s point is very well taken in that we tersely summarize results across several patient scenarios. In the revised manuscript, we now discuss Table 2 results hypothetical patient by hypothetical patient to provide more detail as suggested.*

3) The discussion is unfortunately very disappointing at the moment. It is much too superficial. Important current literature is not discussed. From my point of view, the discussion needs to be rewritten in large parts.

*Response: The reviewer’s point is again well taken. We now summarize the results from this study in the context of systematic reviews (Haiduc et al. 2020 J Cardiac Surg; Ramanathan et al. 2021 Critical Care; Ling et al. 2022 Critical Care) and large multi-site cohort studies (Schmidt Lancet Respiratory Med 2023; Urner BMJ 2022) that show that age and comorbidity are positively associated with mortality.*

4) Likewise, the figures need a complete revision. They are currently not suitable for publication in this form, neither in terms of content nor optically.

*Response: After consultation with the editor, we have been advised to retain the 3 tables as presented in the original submission.*

5) In summary, the publication deals with a very interesting topic. If the authors want to publish this in PLOS ONE, a comprehensive revision is urgently necessary. I am already looking forward to reading this revision.

*Response: We again appreciate the encouraging reveiwer comments and hope that the revision is deemed responsive to comments above.*
